# Exploring the magnetohydrodynamic stretched flow of Williamson Maxwell nanofluid through porous matrix over a permeated sheet with bioconvection and activation energy

**DOI:** 10.1038/s41598-021-04581-1

**Published:** 2022-01-07

**Authors:** Sohaib Abdal, Imran Siddique, Dalal Alrowaili, Qasem Al-Mdallal, Sajjad Hussain

**Affiliations:** 1 Northwest University, School of Mathematics, Xian, 7100069 China; 2 Department of Mathematics, University of Management and Technology, Lahore, 54770 Pakistan; 3 Department of Mathematics, College of Science, Jouf University, Sakaka, 2014 Saudi Arabia; 4 Department of Mathematics Sciences, UAE University, Al Ain, 15551 United Arab Emirates; 5 School of Aerospace and Mechanical Engineering, Nanyang Technological University, Singapore, Singapore

**Keywords:** Engineering, Mathematics and computing

## Abstract

The evolution of compact density heat gadgets demands effective thermal transportation. The notion of nanofluid plays active role for this requirements. A comparative account for Maxwell nanofluids and Williamson nanofluid is analyzed. The bioconvection of self motive microorganisms, non Fourier heat flux and activation energy are new aspects of this study. This article elaborates the effects of viscous dissipation, Cattaneo–Christov diffusion for Maxwell and Williamson nanofluid transportation that occurs due to porous stretching sheet. The higher order non-linear partial differential equations are solved by using similarity transformations and a new set of ordinary differential equations is formed. For numerical purpose, Runge–Kutta method with shooting technique is applied. Matlab plateform is used for computational procedure. The graphs for various profiles .i.e. velocity, temperature, concentration and concentration of motile micro-organisms are revealed for specific non-dimensional parameters. It is observed that enhancing the magnetic parameter *M*, the velocity of fluid decreases but opposite behavior happens for temperature, concentration and motile density profile. Also the motile density profile decrease down for *Pe* and *Lb*. The skin friction coefficient is enhanced for both the Williamson and Maxwell fluid.

## Introduction

In modelling of fluids flows with shear-dependent viscosity, the power-law model is commonly used. But the consequences of elasticity are impossible to be anticipated. The results of elasticity can be achieved by second grade or third grade fluids. But the viscosity is not shear-dependent in these models. In addition, they are unable to determine the impacts of calming stress. A subdivision of fluids of the rate form, viz., Maxwell model can predict stress relaxation and has thus become more common. As in the Maxwell model, a strictly viscous damper and a purely elastic spring can be described. Many researchers have pondered Maxwell nanofluid flow simulations. Maxwell nanofluids with normal kernel through fractional derivatives studded by Abro et al.^1^. Numerical analysis of Maxwell nanofluid flows past a linearly stretched layer was reviewed by Sharama et al.^2^. Assessment of bioconvection in Maxwell nanofluid configured with nonlinear thermal radiation and activation energy using a Riga surface was conducted by Ramesh et al.^3^. Ahmed et al.^4^ reviewed the impact of radiative heat flux in Maxwell nanofluid flow over a chemically reacted spiralling disc. Mathematical analysis of Maxwell nanofluid with hydromagnetic dissipative and radiation was considered by Hussain et al.^5^. Jawad et al.^6^ calculated the entropy generation for magnetohydrodynamic (MHD) mixed convection and Maxwell nano-fluid flow over an elongating and penetrable surface in the presence of heat conductivity, velocity slip boundary condition and thermal radiation.

English scientist Williamson presented the Williamson model^7^ in 1929 and several other researchers studied it. Williamson fluid has a shear thinning property typical of a non-Newtonian fluid model. Later, perturbation solution of incompressible flows in a rock fracturing with a non-Newtonian Williamson fluid was studied by Dapra et al.^8^. Nadeem et al.^9^ studded Wiliamson nanofluid stream on an extended sheet in twenty first century. Even now in 2020, several scholar are rivaling the properties of Williamson nano-fluid such as, analysis of gyrotactic microorganisms with activation energy in the radiated liquid was provoked Haq et al.^10^. The impact of the magnetic field in a curved channel on the peristaltic flow of Williamson fluid was explored by Rashid et al.^11^. Habib et al.^12^ carried out a comparison research on micropolar, Williamson, and Maxwell nanofluids flow owing to a stretched surface in the presence of bioconvection and double diffusion along the activation energy. Waqas et al.^13^ conducted research for the generalized principle of Fourier and Fick to effect Williamson fluid flow. Analysis of heat transfer for electro-osmotic stream across of the micro-channel for Williamson fluid motion was discussed by Noreen et al.^14^.

Hydrodynamic volatility and patterns in suspensions of biased swimming microorganisms are characterized by the term bio-convection. In the early 21st century, biotechnology has developed to incorporate modern and complex disciplines. Bioconvection has various uses in natural systems and biotechnology. Various researchers commit their resources to reveal the characteristics of bio-convection. Bioconvection has various uses in natural systems and biotechnology. Numerical study of bio-convective nanofluid on a magnetohydrodynamic slip flow with stephan blowing was considered by Tuz et al.^15^. The effects of energy on Eyring nanofluid flow containing motile microorganism was studied by Sharief et al.^16^. Sharma et al.^17^ adopted non-dimensional measurements to examine the effect of heat and mass flux on the natural convective laminar movement of a viscoelastic immiscible fluid. Sharma et al.^18^ studied the impacts of a chemical change and a heat source on magneto-hydrodynamic assorted convective mass and heat transfer flow over a vertical plate. For a Casson nanofluid stream over a stretching/shrinking sheet, magagula et al.^19^ tested the influence of an applicable magnetic field, nonlinear thermal radiation and first-order chemical reaction double dispensed bioconvection. Shaw et al.^20^ presented a homogeneous model for oxytactic microbial bioconvection in a non-Darcy permeable material. Khan et al.^21^ studied the impacts of Arrhenius activation energy, binary chemical reaction and viscous dissipation on the bioconvective micropolar flow of nanofluid over a slim moving needle containing gyrotactic micro-organisms.

A highly promising nanostructure analysis was primarily conducted by Choi et al.^22^ to show that the presence of nanoparticles in base fluids can be beneficial in improving the rheological effects of typical base fluids. “Nanofluid is a suspension comprising nanoparticles in a base liquid (water, base fluid mixture, kerosene, bio-fluids and organic liquids) that alters the viscosity, thermal conductivity, density, and mass diffusivity of the base fluid”.In the last few decades, companies have evolved to research fluid mechanics on the nano and micro scales. For improved oil recovery in a heterogeneous two-dimensional anticline geometry, nanofluid flooding investigated by Esfe et al.^23^. Khader et al.^24^utilized the fourth order predictor-corrector finite difference technique to analyse the influence of thermal radiation and a non-uniform heat source on unsteady MHD micropolar fluid flow past a stretching sheet. Verma et al.^25^ examined the steady two-dimensional, laminar, viscous and incompressible boundary layer movement of $Cu/Ag-H_2O$ nanoparticles. kumaran et al.^26^ employed the Keller box technique to examine the effect of thermal conductivity variation and thermal radiation on chemically reacting and free convective Powell-Eyring nanofluid flow across a cylinder. Sharma et al.^27^ explored an irregular magneto-hydrodynamic (MHD) natural convection transmission of mass and thermal over a porous medium sheet under the impact of thermal radiation and thermo-diffusion consequences using the Laplace transform tactic. Jena et al.^28^ numerically investigated nanofluid stream transmission through permeable media with a heat source/sink and a chemical process. Nayak et al.^29^ discussed slip consequences of the flow of chemically reactive Casson nanofluid flowing over an exponentially stretched electromagnetic sheet. Saranya et al.^30^ researched the two-dimensional consistent convective boundary layer fluid motion and heat transmit of Newtonian/non-Newtonian base liquids with magnetic nanoparticles over a flat plate with non-linear thermal radiation and slip implications. Kairi et al.^31^ assumed that the bioconvection of Casson nanoparticles over an inclined elongating sheet. Sharma et al.^32^ exploited the perturbation approach to analyse the electrically conducting fluid motion over a vertical plane sheet. The diverse applications of nanofluids in various aspects are examined by some other scholars^33–36^.

In1942, Hannes Alfvén made the first recorded use of the term “Magnetohydrodynamics”.It originated from the magneto-meaning magnetic field, hydro-meaning water, and dynamics-meaning motion. MHD is utilized in divers fields of engineering, industrial and mostly in the nanotech field. All around the globe, scholars are devoting their time and efforts to reveal the characteristics. In recent time, many scholars worked on the properties of MHD as discussed Rasool et al.^37^. Ahmad et al.^38^ applied the homotopy analysis method (HAM) to examine the fluid film flow of an Oldroyd-B fluid past a spinning disc in three-dimensional space under the implications of heat absorption/omission and radiation terminologies. Gul et al.^39^ employed the RK-4 method to investigate the MHD (magneto-hydrodynamics) unsteady and immiscible flow of nanofluid flow caused by a stretching rotating disc with the effect of Joule heating and dispersion. Gul et al.^40^ investigated the 3-D Darcy-Forchheimer MHD Casson fluid and steady flow between the difference between a disc and a cone in a rotating strategy in the presence of thermal diffusion effect and Brownian movement^41,42^ scrutinized the MHD flow attributes in distinct aspects.

The impact of bioconvection of micro-organism for two different flows of Williamson nanofluid and Maxwell nanofluid is rarely studied in the existing literature. It helps to cope with the possible settling of nano-entities and thus improvement of thermal conductivity of the common fluids can be established. The novelty of this work pertains to Cattaneo–Christov heat flux, bioconvection and activation energy. The buoyancy effects, magnetic field strength imparts differentiated influence on flow temperature and concentration characteristics. The role of these useful and practicable physical aspects for two significant non-Newtonian fluids is enumerated and their comparative outcomes help to broaden our understanding and utilization of these flows. By utilization of similarity transforms enable to yield numerical solution of non-linear coupled system with the implementation of Runge–Kutta method and shooting technique. By utilizing the parametric evolution of bioconvection, nanofluid slips and Cattaneo–Christov factor, the findings are evaluated for Maxwell fluid and Williamson fluid. These results are beneficial for thermal management of heat exchangers of the emerging technologies.

## Physical model and mathematical formulation

**Figure 1:**
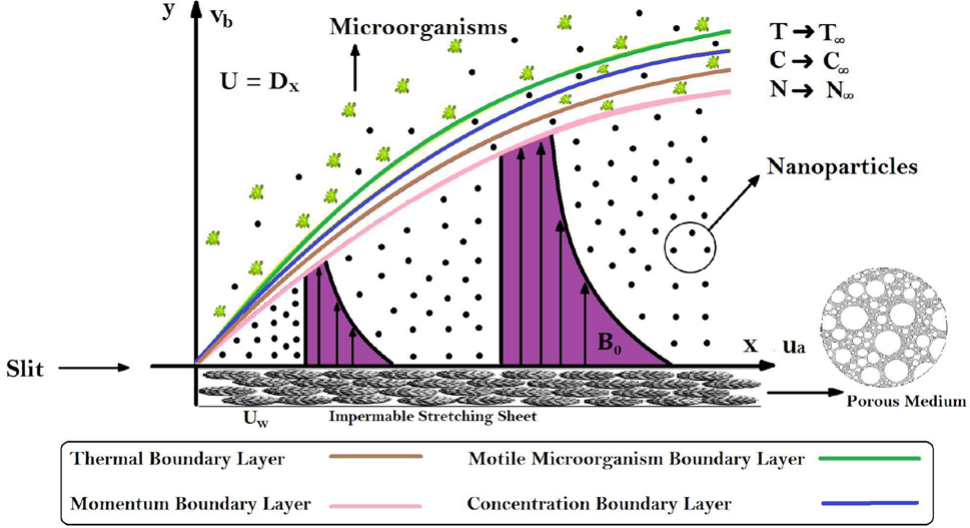
Problem description.

A two-dimensional, steady flow of Maxwell and Williamson nanofluid with activation energy and Cattaneo–Christov diffusion embedded in a porous medium is discussed in the existence of bioconvection of micro-organisms. The fluids flow owing to a stretching sheet, The wall velocity is $U_w = Dx$, *D* is stretching constant. $U_w$ is the stretching velocity of sheet along x-axis. It is assumed that behavior of the flow is linear due to the impermeable stretching sheet. Here $u_a$ and $v_b$ are the velocities of fluids flow along x-axis and y-axis. In Fig. 1, the temperature of fluids, concentration and density of microorganisms near the sheet are taken as *T*, *C*, and *N*. Now our problem represents the basic equations of continuity, equation of momentum, energy equation, concentration equation and gyrotactic microorganism’s concentration equation in Cartesian coordinates.

The following equation are established in the aspects of flow of fluids. The governing equations for the problems are given below^5,8,16^:

1 $$\begin{aligned}&\frac{\partial u_a}{\partial x}+\frac{\partial v_b}{\partial y}=0, \end{aligned}$$

2 $$\begin{aligned} & u_{a} \frac{{\partial u_{a} }}{{\partial x}} + v_{b} \frac{{\partial u_{a} }}{{\partial y}} = \nu \frac{{\partial ^{2} u_{a} }}{{\partial y^{2} }} + \sqrt 2 \nu \Gamma \frac{{\partial u_{a} }}{{\partial y}}\frac{{\partial ^{2} u_{a} }}{{\partial y^{2} }} - \frac{{\sigma B_{0}^{2} u_{a} }}{\rho } - \frac{\nu }{{k^{\prime } }}u_{a} - \lambda _{1} \left[ {u_{a}^{2} \frac{{\partial ^{2} u_{a} }}{{\partial x^{2} }} + v_{b}^{2} \frac{{\partial ^{2} u_{a} }}{{\partial y^{2} }} + 2u_{a} v_{b} \frac{{\partial ^{2} u_{a} }}{{\partial y\partial x}}} \right] \\ & \;\;\; + \;\left( {\frac{1}{\rho }} \right)\left( {(1 - C_{\infty } )\rho \beta (T - T_{\infty } ) - (\rho _{p} - \rho )g(C - C_{\infty } ) - (N - N_{\infty } )g\gamma (\rho _{m} - \rho )} \right), \\ \end{aligned}$$

3 $$\begin{aligned}&u_a\frac{\partial T}{\partial x}+v_b\frac{\partial T}{\partial y}=\alpha \frac{\partial ^2 T}{\partial y^2}+\frac{\rho _p C_p}{\rho C} \bigg [D_B\frac{\partial C}{\partial y}\frac{\partial T}{\partial y}+\frac{D_T}{T_\infty }(\frac{\partial T}{\partial y})^2 \bigg ] \nonumber \\&\quad + \, \tau _1 \bigg [u_a \frac{\partial u_a}{\partial x} \frac{\partial T}{\partial x}+v_b \frac{\partial v_b}{\partial x} \frac{\partial T}{\partial x} + u_a \frac{\partial v_b}{\partial x} \frac{\partial T}{\partial x}+v_b \frac{\partial u_a}{\partial y} \frac{\partial T}{\partial x}+2u_a v_b\frac{\partial ^2 T}{\partial x \partial y}+u_a^2 \frac{\partial }{\partial x}\frac{\partial T}{\partial x}+v_b^2 \frac{\partial }{\partial y}\frac{\partial T}{\partial y} \bigg ], \end{aligned}$$

4 $$\begin{aligned}&u_a\frac{\partial C}{\partial x}+v_b\frac{\partial C}{\partial y}=D_B\frac{\partial }{\partial y}\frac{\partial C}{\partial y} + \frac{D_T}{T_\infty } \frac{\partial }{\partial y}\frac{\partial T}{\partial y} - (Kr)^2 (C_w-C_\infty ) \bigg (\frac{T}{T_\infty } \bigg )^m exp \bigg (\frac{-E_a}{k_2 T} \bigg ), \end{aligned}$$

5 $$\begin{aligned}&u_a\frac{\partial N}{\partial x}+ v_b\frac{\partial N}{\partial y} + b W_c \frac{\partial }{\partial y} \bigg (\frac{n}{\Delta C}\frac{\partial C}{\partial y} \bigg )=D_m \frac{\partial }{\partial y}\frac{\partial N}{\partial y}. \end{aligned}$$

6 $$\begin{aligned}&{\left. \begin{aligned} {u_a} ={U_w = Dx}, {v_b}={v}_w, {T}-{T}_w(x)=0, {C}-{C}_w(x)=0, {N}-{N}_w(x)=0, \quad at\quad y=0,\\ {u_a}\rightarrow 0, \quad {T}\rightarrow {T}_\infty ,\quad {C}\rightarrow {C}_\infty , \quad {N}\rightarrow {N}_\infty \quad as\quad y \rightarrow \infty . \end{aligned}\right\} } \end{aligned}$$

Using the similarity variables:

7 $$\begin{aligned} {\left. \begin{aligned} \eta = \sqrt{\frac{D}{\nu }}y, \ u_a = Dxf'(\eta ), v_b = -\sqrt{{D\nu }}f(\eta ), \theta (\eta ) = \frac{T-T_\infty }{T_w - T_\infty },\\ \phi (\eta ) = \frac{C-C_\infty }{C_w - C_\infty },\chi (\eta ) = \frac{N-N_\infty }{N_w - N_\infty } \end{aligned}\right\} } \end{aligned}$$

where we have $\lambda _1$ is fluid relaxation time, $\Gamma$ is Williamson fluid parameter. It is to mentioned that in Eq. (2) when $\lambda _1 = 0$ and $\Gamma \ne 0$, it is Williamson fluid flow. Also for $\Gamma = 0$ and $\lambda _1 \ne 0$, than it is Maxwell fluid flow. Equation (1) is balanced identically. Equations (2) to (5) are rescaled as below:

8 $$\begin{aligned}& f^{\prime \prime \prime} - f^{\prime 2} + ff^{\prime \prime} - \beta (f^2f^{\prime \prime \prime}-2ff^{\prime }f^{\prime \prime }) + \lambda f^{\prime \prime }f^{\prime \prime \prime} - (M+K_p)f^{\prime } + \omega (\theta - Nr\phi - Rb\chi ) = 0,\end{aligned}$$

9 $$\begin{aligned}&\theta ^{\prime \prime} + Pr f\theta ^{\prime } + \frac{Nc}{Le}\theta ^{\prime } \phi ^{\prime } + \frac{Nc}{Le * Nbt}\theta ^{\prime 2} + b (f^2 \theta ^{\prime \prime } + ff ^{\prime }\theta ^{\prime }) = 0,\end{aligned}$$

10 $$\phi ^{{\prime \prime }} + Sc(f\phi ^{\prime } ) + \left( {\frac{1}{{Nbt}}} \right)\theta ^{{\prime \prime }} - Sc*A\phi (1 + \delta \theta )^{m} \exp \left( {\frac{{ - E}}{{1 + \delta \theta }}} \right) = 0,$$

11 $$\begin{aligned}&\chi ^{\prime \prime } +Lb(f\chi ^{\prime })-Pe[\phi ^{\prime \prime }(\chi +\Omega )+\chi ^{\prime } \phi ^{\prime }] = 0.\end{aligned}$$

12 $$\begin{aligned}&{\left. \begin{aligned} f(0) = S, \ f'(0) = 1, \ \theta (0)=1,\ \phi (0)=1,\ \chi (0)=1, \ at \ \eta = 0,&\\ f'(\infty )\rightarrow 0, \ \theta (\infty )\rightarrow 0, \phi (\infty )\rightarrow 0, \chi (\infty )\rightarrow 0, \ as \ \eta \rightarrow \infty .&\end{aligned}\right\} } \end{aligned}$$

The non-dimensional parameters in their respective order:

$\beta$, $\lambda$ *M*, *Kp*, $\omega$, *Nr*, *Rb*, *Rd*, *Pr*, *Le*, *Nbt*, *b*, *Q*, *Sc*, *A*, $\delta$, *E*, *Lb*, *Pe*, $\Omega$, and *S* are Deborah number, non-newtonian Williamson parameter, magnetic parameter, porosity parameter, mixed convection, buoyancy ratio, Rayleigh number, radiation, Prandtl number, Lewis number, diffusivity ratio, thermal relaxation constant, heat source, Schimdt number, dimensionless reaction rate, temperature difference, activation energy, bio-convection Lewis number, bio-convection Peclet number, microorganism concentration difference, suction/injection parameter.

Where: $$\beta = \lambda _1 D$$. $$M = \frac{\sigma B_0^2}{D \rho }$$, $$Kp = \frac{\nu }{k_1 D}$$, $$\omega = \frac{\beta _1 g (1-C_\infty )(T_w-T_\infty )}{D^2 x}$$, $$Nr = \frac{(\rho _p - \rho )(C_w-C_\infty )}{\beta _1 \rho (1-C_\infty )(T_w-T_\infty )}$$, $$Rb = \frac{(\rho _m - \rho )\gamma N_\infty }{(1-C_\infty )\rho \beta T_\infty }$$, $$Rd = \frac{16 T^3_\infty \sigma ^*}{3k^* \kappa }$$, $$Pr = \frac{\nu }{\alpha }$$, $$Le = \frac{\alpha }{D_B}$$, $$Nbt = \frac{D_B T_\infty (C_w - C_\infty )}{D_T (T_w - T_\infty )}$$, $$b = \tau _1 D$$, $$Sc = \frac{\nu }{D_B}$$, $$A = \frac{k^2r}{D}$$, $$\delta = \frac{T_w - T_\infty }{T_\infty }$$, $$E = \frac{E_a}{k_2 T_\infty }$$, $$Lb = \frac{\nu }{D_m}$$, $$Pe = \frac{b_1 Wc}{D_m}$$, $$\Omega = \frac{N_\infty }{N_w - N_\infty }$$, $$S = \frac{-v_w}{\sqrt{D \nu }}$$.

The physical quantities are stated as^43^:

$Cf_x$ (skin friction coefficient), $Nu_x$ (local Nusselt number), $Sh_x$ (local Sherwood number) and $Nn_x$ (local density of microorganism) are given below:

13 $$\begin{aligned} {\left. \begin{aligned} Cf_x = \frac{\tau _w}{\rho U^2w},\ Nu_x = \frac{xq_w}{k(T_w - T_\infty )},&\\ Sh_x = \frac{xq_m}{D_B(C_w - C_\infty )},\ Nn_x = \frac{xq_n}{D_m(N_w - N_\infty )}.&\end{aligned}\right\} } \end{aligned}$$

where $\tau _w$, $q_w$, $q_m$ and $q_n$ denotes shear stress, surface heat flux, surface mass flux and motile microorganism flux are given by (at $y =0$),

14 $$\begin{aligned} {\left. \begin{aligned} \tau _w = \mu (1 + \beta )(1 + \frac{\Gamma }{2}\frac{\partial u_a}{\partial y}) \frac{\partial u_a}{\partial y},\ q_w = -K \frac{\partial T}{\partial y},&\\ q_m = -D_B \frac{\partial C}{\partial y},\ q_n = -D_m \frac{\partial N}{\partial y}.&\end{aligned}\right\} } \end{aligned}$$

On solving these quantities with the help of given similarity transformation, we obtain:

15 $$\begin{aligned} {\left. \begin{aligned}&C_f(Re_x)^{-1/2} = (1 + \beta )(f^{\prime \prime }(0) + \frac{\lambda }{2}f^{\prime \prime }(0)^2),\ Nu_x(Re_x)^{-1/2} = -\theta ^{\prime }(0),\\&Sh_x(Re_x)^{-1/2} = -\phi ^{\prime }(0),\ Nn_x(Re_x)^{-1/2} = -\chi ^{\prime }(0). \end{aligned}\right\} } \end{aligned}$$

where, $(Re_x) = \frac{x U_w}{\nu }$ is the local Reynolds number.

## Solution procedure

For numerical results, nonlinear ordinary differential Equations (8) to (11) with the given boundary conditions Eq. (12) are solved by Runge–Kutta method of order four along with shooting technique. To execute this numerical method, the new variable expressed as below^44–46^:

16 $$\begin{aligned} {\left. \begin{aligned}&s_1^{\prime } = s_2 \\&s_2^{\prime } = s_3 \\&s_3^{\prime } = s^2_2 - s_1 s_3 + \beta (s_1^2 ds_3-2 s_1 s_2 s_3) - \lambda s_3 ds_3 + (M+Kp)s_2 - \omega (s_4 - Nr s_6 - Rb s_8) \\&s_4^{\prime } = s_5 \\&s_5^{\prime } = -Pr s_1 s_5 - \frac{Nc}{Le} s_5 s_7 - \frac{Nc}{Le*Nbt} s^2_5 - b(s^2_1 ds_5 +s_1 s_2 s_5) \\&s_6^{\prime } = s_7 \\&s_7^{\prime } = -Sc s_1 s_7 - \frac{1}{Nbt} ds_5 + Sc*A[1+\delta s_6]^n exp[\frac{-E}{1+\delta s_6}] s_8 \\&s_8^{\prime } = s_9 \\&s_9^{\prime } = -Lb s_1 s_9 + Pe[s_7 s_9 + (\Omega + s_8) ds_7] \end{aligned}\right\} } \end{aligned}$$

along with the boundary conditions^47^:

17 $$\begin{aligned} {\left. \begin{aligned}&s_1=S, \ s_2=1,\ s_4=1 ,\ s_6=1,\ s_8=1, at \eta =0,\\&s_2 \rightarrow 0,\ s_4 \rightarrow 0,\ s_6 \rightarrow 0,\ s_8 \rightarrow 0 \ as \ \eta \rightarrow \infty . \end{aligned}\right\} } \end{aligned}$$

## Results and discussions

In this part, we presented and explained the outcomes as computed from the above mentioned procedure. The validation of current results is established when these are compared with the existing findings of^48–50^ in limiting cases.The four sets of results are contained in Table 1 and a close agreement is seen among them. Table 2 indicates that the drag force is supplemented with incremental variation of *M*, $K_p$. *Nr*, *Rb* in case of Maxwell fluid $(\beta = 0.5)$ as well as Williamson fluid $(\lambda = 0.1)$ and hence the magnitude of $-f''(0)$ (skin friction factor) is enhanced because of the additional resistance to the flow that comes into play with enhancement of magnetic parameters. However, the skin friction is reciprocated against mixed convection parameter $\omega$, because the rise in mixed convection helps to accelerate the flow. Further, it is seen that the absolute value of $-f''(0)$ is larger for Maxwell nanofluid than that of Williamson nanofluid. Table 3 shows that Nusselt number $-\theta '(0)$ improves in direct relation to *Pr*, *Nbt* and *b*, but it diminishes against *Nc*. Table 4 convinced that $-\phi '(0)$ recedes only when *E* rises, while $-\phi '(0)$ is upsurged directly with *Sc*, *A*, $\delta$ and *n*. From Table 5, it is noticed that magnitude of motile density $-\chi '(0)$ is increased when *Lb*, *Pe* and $\Omega$ are uplifted. Also, it is observed that Maxwell nanofluid takes larger values than that of Williamson nanofluid for Nusselt number $-\theta '(0)$, $-\phi '(0)$ and for motile density $-\chi '(0)$.

**Table 1:** The comparative outputs for $-\theta '(0)$.

*Pr*	Nadeem et al.^48^	Khan and Pop^49^	Golra and Sidawi^50^	Present results
0.07	0.066	0.066	0.066	0.065
0.2	0.169	0.169	0.169	0.167
0.7	0.454	0.454	0.454	0.435
2.0	0.911	0.911	0.911	0.910

**Table 2:** Results for $-f''(0)$.

*M*	*Kp*	${\varvec{\omega }}$	*Nr*	*Rb*	Maxwell fluid $\beta = 0.5$	Williamson fluid $\lambda = 0.1$
0.1	0.1	0.1	0.1	0.1	2.5113	1.2342
0.3					2.6257	1.3090
0.5					2.7341	1.3796
0.5	0.2				2.5693	1.2722
	0.4				2.6806	1.3448
	0.5				2.7341	1.3796
	0.5	0.1			2.7341	1.3796
		0.2			2.6512	1.3339
		0.3			2.5712	1.2897
		0.1	0.1		2.7341	1.3796
			0.2		2.7407	1.3834
			0.3		2.7474	1.3873
			0.1	0.1	2.7341	1.3796
				0.2	2.7389	1.3824
				0.3	2.7438	1.3852

**Table 3:** Results for $-\theta '(0)$.

*Pr*	*Nc*	*Nbt*	*b*	Maxwell fluid $\beta = 0.5$	Williamson fluid $\lambda = 0.1$
0.71	0.5	2.0	0.5	0.4045	0.4418
1.0				0.5237	0.5680
1.5				0.7129	0.7639
0.71	0.1			0.4439	0.4843
	0.5			0.4045	0.4418
	1.0			0.3598	0.3934
	0.5	1.0		0.3942	0.4306
		2.0		0.4045	0.4418
		3.0		0.4081	0.4456
		2.0	0.1	0.3769	0.4063
			0.3	0.3905	0.4238
			0.5	0.4045	0.4418

**Table 4:** Results for $-\phi '(0)$.

*Sc*	*A*	${{\delta }}$	*E*	n	Maxwell fluid $\beta = 0.5$	Williamson fluid $\lambda = 0.1$
1.0	0.1	0.1	0.3	0.5	0.7333	0.7408
2.0					1.0157	1.0395
3.0					1.2887	1.3218
2.0	0.1				1.0157	1.0395
	0.2				1.1912	1.2084
	0.3				1.3389	1.3526
	0.1	0.1			1.0157	1.0395
		0.2			1.0261	1.0493
		0.3			1.0359	1.0586
		0.1	0.1		1.0546	1.0767
			0.3		1.0157	1.0395
			0.5		0.9818	1.0073
			0.3	0.1	1.0101	1.0342
				0.3	1.0129	1.0369
				0.5	1.0157	1.0395

**Table 5:** Results for $-\chi '(0)$.

*Lb*	*Pe*	${{\Omega }}$	Maxwell fluid $\beta = 0.5$	Williamson fluid $\lambda = 0.1$
0.4	1.2	0.2	1.6319	1.6748
0.8			1.7782	1.8269
1.2			1.9140	1.9662
1.2	0.4		1.0467	1.0892
	0.8		1.4761	1.5228
	1.2		1.9140	1.9662
	1.2	0.1	1.8102	1.8621
		0.2	1.9140	1.9662
		0.3	2.0178	2.0704

**Figure 2:**
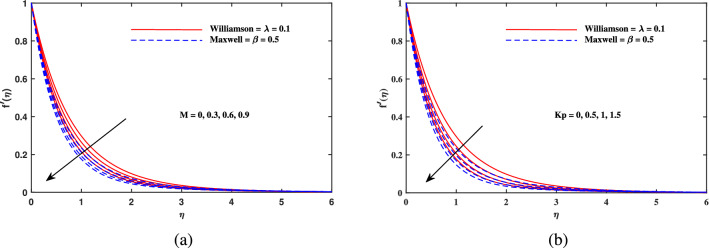
Velocity $f'(\eta )$ fluctuation with (**a**) *M* and (**b**) $K_p$.

**Figure 3:**
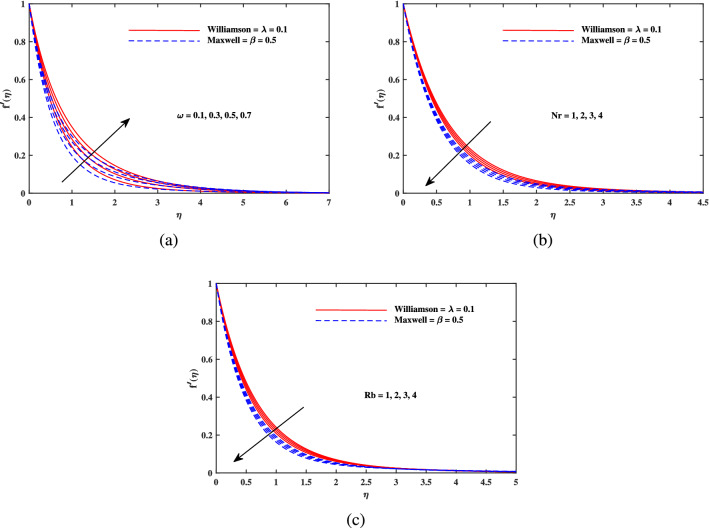
Velocity $f'(\eta )$ fluctuation with (**a**) $\omega$, (**b**) *Nr* and (**c**) *Rb*.

**Figure 4:**
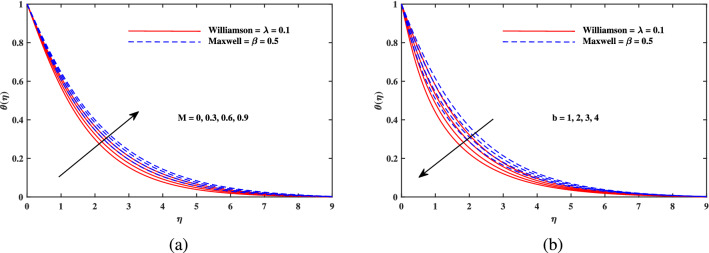
Temperature $\theta (\eta )$ fluctuation with of (**a**) *M* and (**b**) *b*.

**Figure 5:**
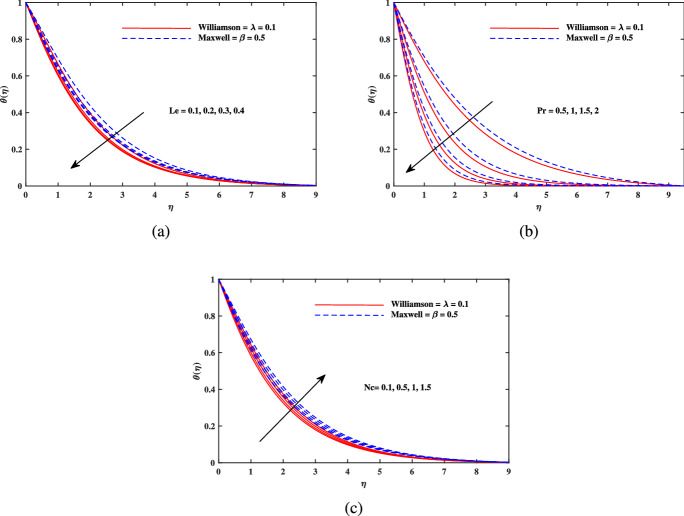
Temperature $\theta (\eta )$ fluctuation with (**a**) *Le*, (**b**) *Pr* and (**c**) *Pr*.

**Figure 6:**
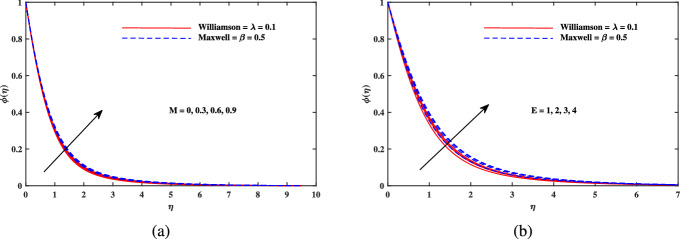
Concentration $\phi (\eta )$ fluctuation with (**a**) *M* and (**b**) *E*.

**Figure 7:**
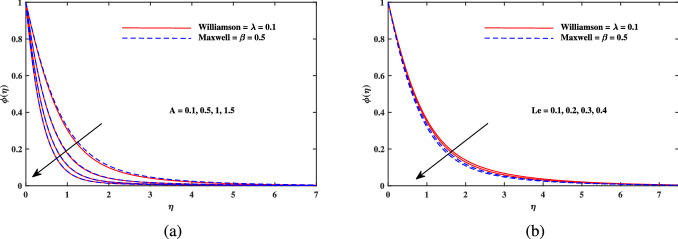
Concentration $\phi (\eta )$ fluctuation with (**a**) *A* and (**b**) *Le*.

**Figure 8:**
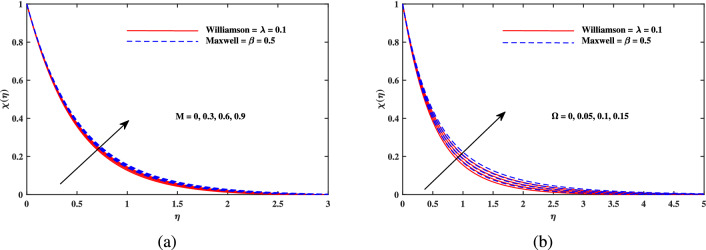
Motile density $\chi (\eta )$ fluctuation with (**a**) *M* and (**b**) $\omega$.

**Figure 9:**
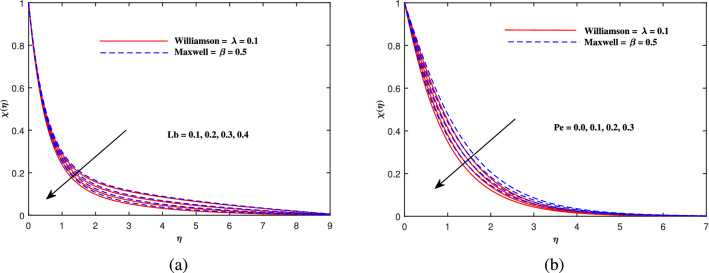
Motile density $\chi (\eta )$ fluctuation with (**a**) *Lb* and (**b**) *Pe*.

Figure 2a explains the effect of magnetic field *M* on velocity profile $f'(\eta )$ for Maxwell and Williamson fluids. A reduction is produced in the boundary layer thickness by increasing the value of *M*. It is observed that Williamson fluid is highly affected as compared to the Maxwell fluids. Physically, the magnetic field has ability to enhance the drag force which is said as Lorentz force. This force prevents the fluid from flowing as well as causes an increment in the thickness of boundary layer. From Fig. 2b, it is seen that the enhanced porosity parameter $K_p$ retards the flow velocity. Because $K_p$ has ability to produce resistance for fluid flow. Mixed convection parameter effect on $f'(\eta )$ for Williamson and Maxwell nanofluids is discussed in Fig. 3a. The greater values of mixed convection parameter $\omega$ causes an increment in $f'(\eta )$ due to the larger buoyancy force. Figure 3b and c both explain the aspects of (*Rb*) bioconvection Rayleigh number as well as (*Nr*) buoyancy ratio parameter on $f'(\eta )$. The changing values of both the parameters cause decline in velocity distribution significantly. Physically, both parameters show their relation with buoyancy ratio forces which causes resistance in the motion of the two fluids. Moreover, the velocity for Williamson nanofluids is faster than that of Maxwell nanofluids. It means Maxwell fluid imparts more viscus effects on the flow than the Williamson fluid. The impacts of magnetic parameter *M* and Cattaneo-Christov parameter *b* on temperature profile $\theta (\eta )$ are illustrated in Fig. 4. Physically, *M* has the ability to intensify the temperature distribution profile due to the involvement of Lorentz force. Moreover by increasing the Cattaneo-Christov parameter *b* causes a reduction in $\theta (\eta )$. Thus Maxwell fluid diffuses more heat as compared to Williamson fluid and hence the temperature curve for Maxwell fluid is elevated than that of Williamson fluid. As it is clear that $\lambda$ is a ratio between buoyancy forces to viscous force. Due to which a little change in $\lambda$ causes a decrement in the $\theta (\eta )$. The impact of Lewis number *Le* on $\theta (\eta )$ is depicted in Fig. 5a. Depreciation in temperature profile is due to its direct relation with diffusivity of mass which causes reduction in the temperature distribution. The effect of *Pr* can be visualized on temperature profile in Fig. 5b. It is clear that by increasing the values of *Pr* causes a reduction in $\theta (\eta )$. Physically, it is due to the reverse relation of *Pr* with the thermal diffusivity. Fig. 5c explains the effect of *Nc* on temperature distribution, an increment in *Nc* causes an accession in temperature distribution. From Fig. 6, it can be visualized that increment in the magnetic number *M* as well as activation energy *E* causes an accession in the concentration profile $\phi (\eta )$. Increment in *M* causes an increase in Lorentz force which causes more resistance to the flow of Williamson fluid as compared to Maxwell fluid. A decrease in concentration profile $\phi (\eta )$ can be observed in Fig. 7 with the exceeding of chemical reaction rate parameter *A* as well as Lewis number *Le*. The larger inputs of *A* means faster chemical reaction to decline the concentration function $\phi (\eta )$. Similarly, higher Lewis number *Le* causes depreciation in $\phi (\eta )$ because it is related reciprocally to mass diffusivity. While the increment in *A* generates more resistance in Williamson fluid flow as compared to Maxwell fluids. Moreover decrement in mass diffusivity becomes Williamson fluid less resistive as compared to Maxwell fluid for flow. The motile density profile $\chi (\eta )$ is increased with the increment in *M*. It is due to slowing of flow. The curves generated by *M* results an accession in the motile density profile $\chi (\eta )$. It also shows an accession in it by increasing the value of microorganism difference parameter $\Omega$ which can be observed in Fig. 8. Figure 9 is drawn to discuss the results of Lewis number *Lb* as well as Peclet number *Pe*. The diffusivity of microorganisms is related with Peclet number *Pe*, which means greater value of Peclet number causes depreciation in diffusivity of microorganisms. The increment in the Peclet number Pe starts a reduction in the motile organisms boundary layer thickness, as well as a decrement in motile density profile $\chi (\eta )$. Moreover, it is clear that larger value of *Lb* causes a lesser microorganism distribution $\chi (\eta )$.

## Conclusion

This article explores the effect of magnetic field, bio-convection and activation energy for Williamson and Maxwell nanofluid on a porous stretched sheet with Cattaneo–Christov diffusion. The main findings of this study are given below:

- Enhancement in magnetic parameter *M* retards the flow of Williamson fluid and Maxwell fluids.
- Rayleigh number *Rb*, buoyancy ratio parameter *Nr*, mixed convection parameter $\omega$, and *Kp* parameters highly influence on Williamson fluid than Maxwell fluid.
- For temperature profile Cattaneo Christov parameter *b*, Lewis number *Le*, and Prandtl number *Pr* have similar effects. Maxwell fluid is more affected by these parameters than Williamson fluid. On contrary, *M* and *Nc* shows opposite behavior for it.
- For concentration profile Williamson fluid is more influenced than Maxwell fluid by *M* as compared to the other various parameters i.e. Activation energy *E*, *A* and *Le*.
- For motile density profile various parameters i.e. *M*, microorganisms difference parameter $\Omega$, *Lb* and Peclet number *Pe* express more effect for Maxwell fluids as compared to Williamson fluids.
- Skin friction coefficient enhanced for $\beta$, *M*, $K_p$, *Nr* and *Rb* while decrease down for $\lambda$ and $\omega$.
- Nusselt number reduces when uplifting the parameter *Nc*, while it increases for *Pr*, *Nbt*, and *b*.
- Sherwood number increases for *Sc*, *A*, $\delta$ and *n* while it reduces when uplifting the parameter *E*.
- Motile density number increases for *Lb*, $\Omega$ and *Pe*.

## Future direction

These results can be further modified and improved for flow of hybrid nanofluids with the implementation of finite element or finite difference discretization.

## Latin symbols

*u*, *v*: Velocity components
(x,y): Cartesian coordinates
$u_w$: Velocity of the fluid at wall
*n*: Power law index
$B_o$: Magnetic field strength
*C*: Concentration of nanoparticles
*T*: Temperature of nanoparticles
*N*: Micro-rotation vector
$k^*$: Mean absorption co-efficient
$k_1$: Permeability of porous medium
*k*: Fluid parameter
$C_p$: Specific heat at constant pressure
$\tau _1$, $\tau _2$: Relaxation time of heat flux
$D_B$: Brownian diffusion constant
$D_T$: Thermophoresis diffusion coefficient
$T_{\infty }$: Free stream temperature
$C_{\infty }$: Free stream concentration of nanoparticles
$Q_1$: Heat source/sink coefficient
*M*: Magnetic field parameter
*K*: Micropolar parameter
*Kp*: Porosity parameter
*t*: Time
*Pr*: Prandtl number
*R*: Thermal radiation parameter
*Nb*: Brownian motion parameter
*Nt*: Thermophoresis parameter
*Sc*: Schmidt number
$Q'$: Heat source parameter
$T_w$: Wall constant temperature
$C_w$: Nanoparticles concentration at wall
$W_e$: Local Weissenberg number
*f*: Dimensionless stream function
$C_f$: Skin friction coefficient
$$Sh_x$$: Sherwood number
$$Nu_x$$: Nusselt number
$$Re_x$$: Local Reynolds

## Greek symbols

$\alpha$: Thermal diffusivity of base fluid
$\mu$: Viscosity of fluid
$$\psi$$: Stream function
$$\nu$$: Kinematic viscosity
$$\sigma$$: Electrical conductivity
$\sigma ^*$: Stefan–Boltzmann constant
$\rho$: Density of fluid
$$\rho _p$$: Nanoparticle’s density
$\gamma$: Spin gradient viscosity
$\gamma _1$: Thermal relaxation parameter
$\gamma _2$: Solutal relaxation parameter
$$\eta$$: Similarity variable
$$\theta$$: Similarity temperature
$\phi$: Similarity concentration of nanoparticles
$\Gamma$: Material time constant
$$\delta$$: Unsteadiness parameter

## Subscripts

*p*: Nanoparticles
*w*: On the accelerated surface
$$\infty$$: Free stream
